# Between regulatory functions and emotional burden: balancing engagement in digital health interventions for self-care in chronic illness

**DOI:** 10.3389/fpsyg.2025.1685934

**Published:** 2025-11-28

**Authors:** Daniela Lemmo, Roberto Bianco, Fabrizio Mezza, Vincenzo De Luca, Maddalena Illario, Guido Iaccarino, Maria Francesca Freda

**Affiliations:** 1 Department of Humanities, University of Naples Federico II, Naples, Italy; 2 Department of Mental, Physical Health and Preventive Medicine, University of Campania Luigi Vanvitelli, Caserta, Italy; 3 Department of Public Health, University of Naples Federico II, Naples, Italy; 4 Department of Clinical Medicine and Surgery, University of Naples Federico II, Naples, Italy; 5 Interdepartmental Research Center for Hypertension and Related Conditions, University of Naples Federico II, Naples, Italy

**Keywords:** engagement, digital health interventions, self-care, hypertension, reflexive thematic analysis, clinical psychology

## Abstract

**Introduction:**

Digital Health Interventions (DHIs) are increasingly used to support self-care in chronic illness. The clinical utility of these technologies for health is well established, but there is limited understanding of psychological and emotional processes that sustain long-term engagement with these technologies. This qualitative study investigates how individuals with hypertension experience and attribute meaning to their engagement with digital self-monitoring tools.

**Methods:**

McCarron’s engagement model—specifically the “retain” phase—and Braun and Clarke’s reflexive thematic analysis were used to analyze 35 semi-structured interviews with adults who used ICT-enabled blood pressure monitors and smart scales.

**Results:**

In the retain phase, engagement in DHIs is characterized by 3 main themes: (1) Reassurance and Sense of Control Over Health Status through Digital Tools; (2) Engaged but Ambivalent and Anxious: The Emotional Cost of Monitoring; (3) Connected Through Data as Numbers Redefine the Therapeutic Dialog.

**Discussion:**

From the results we identified five psychological functions of engagement with digital monitoring tools, each showing both functional and non-functional or ambivalent expression. Engagement with DHIs cannot be reduced to simple observable behavior, but must be understood as a dynamic, iterative and situated process, supported by the regulatory functions in the context of self-care. Clinicians, psychologists, and designers are therefore called upon to recognize and value the psychological functions implicit in DHIs in order to promote truly sustainable, meaningful, and person-centered care practices and engagement.

## 1 Introduction

Healthcare systems worldwide face increasing pressure from chronic illnesses so public health policies now focus on self-care as the central element of assistance. The management of chronic conditions as hypertension, diabetes and cardiovascular diseases through continuous self-care activities helps patients avoid complications and achieve their best health outcomes. Digital Health Interventions (DHIs) including mobile apps and wearable technologies and remote monitoring systems have proven to be effective tools for condition management. ICT-enabled monitoring tools have received significant attention because they show promise for better patient engagement and improved health results and mental wellness support. The technologies serve dual purposes of clinical outcome enhancement and behavioral modification and patient autonomy development and healthcare provider-patient communication improvement (Huckvale et al., 2019; World Health Organization, 2020; Forman et al., 2016; Möller et al., 2017). DHIs integrate into patients’ daily activities to create new opportunities for illness management in their daily lives. A systematic review by Bashi et al. (2017) shows that DHIs effectively enhance health results through their ability to track patients remotely and improve medical staff communication and helping patients stick to their treatment plans.

Smartwatches and smart scales enable people to track their cardiovascular health and weight management which helps them make better lifestyle choices according to Yen and Huang (2022). Healthcare providers can access patient data in real-time or remotely through these integrated technologies which enables them to modify treatment plans according to patient needs (Liu et al., 2020). Digital devices enable self-care processes that transform traditional medical prescription adherence into an advanced practice which combines body monitoring with self-awareness and personal drive and decision-making abilities (Riegel et al., 2012). Digital engagement demands more than behavioral adherence because it creates narrative challenges that affect how people understand illness and care while they establish their identity through technology-mediated interactions with themselves and their healthcare providers.

From a clinical psychology perspective, self-care is built and renewed over time, through the meaning attributed to the experience of illness, the sense of competence, and the willingness to change that must be maintained over time (Lemmo et al., 2025b; Lemmo et al., 2025a). From this perspective, digital technologies can become powerful tools not only to inform, but to transform the way people perceive themselves with respect to their health and the healthcare system. In this regard, the *Uses and Gratifications Theory* (Blumler and Katz, 1974; Papacharissi and Rubin, 2000) highlights how the way these tools are used can profoundly impact psychological well-being, as people seek answers to emotional, cognitive, and social needs in digital media (Whiting and Williams, 2013). The implementation of these technologies functions as an emotional regulation system, which helps users manage their disturbing emotional states (Brimmel et al., 2023; Wolfers and Schneider, 2021). However, these psychological benefits may be temporary, as their affective-regulation function might not persist over time—highlighting the need to investigate users’ subjective experiences. While devices provide structured continuity, they can also generate emotional tension and dependence among users. The effectiveness of DHIs in supporting self-care remains limited because users do not engage with these tools sufficiently (Baumel et al., 2019; Birnbaum et al., 2015; Meyerowitz-Katz et al., 2020; Pratap et al., 2020; Torous et al., 2020; Yeager and Benight, 2018; Serlachius et al., 2019). Digital interventions require basic user engagement to deliver their intended benefits, yet in literature, engagement is often measured through simple metrics of usage, duration and frequency, without taking into account the psychological and relational aspects (Kelders et al., 2020; Short et al., 2018). From this perspective, engagement becomes a behavioral outcome, without any in-depth analysis of the underlying mechanisms. Even the most widespread theoretical models, such as UTAUT (Venkatesh et al., 2003), fail to include the nuances of the emotional, symbolic, and relational meanings of long-term involvement in chronic care. More recent models, such as DIEGO (O’Connor et al., 2016), propose an understanding of the factors affecting patient and public engagement and enrollment in consumer digital health, emphasizing the central role of motivation, agency, and adaptation to the context. A challenge for clinical psychology, therefore, remains understanding what “being involved” really means. As shown by Ng et al. (2019), the lack of a shared definition makes it difficult to interpret the results of studies. Engagement, then, should be understood as an evolving psychological process, modulated by the way each individual makes sense of the illness and their role in care (PHE model, Graffigna and Barello, 2017). The phase model proposed by McCarron et al. (2020) helps us understand this complexity by distinguishing between the phases of “recruit,” “retain,” and “sustain”: from the initial decision to engage, to long-term maintenance, up to long-term sustainability. However, each phase brings with it potential ambivalence. The ability to track data brings comfort to some patients (Morton et al., 2022) but monitoring creates stress for others which intensifies the conflict between self-reliance and dependence (Schermer, 2009). The clinical psychology perspective demands an understanding of engagement as an ongoing process that exists within specific contexts (Lemmo et al., 2025b; Lemmo et al., 2025a) because it cannot be simplified to behavioral implementation or digital usage frequency. The ways in which people relate to healthcare devices are varied: for some, it is a reliable companion that offers containment and structure; for others, it is a constant reminder of the illness.

Psychodynamic and sociological theories also suggest that digital monitoring can alter the way individuals perceive their bodies and position themselves within the therapeutic relationship (Lupton, 2013, 2014). Patients may come to attribute to these technologies’ functions of care, control, or even judgment, reflecting the emotional complexity of their interactions with digital tools. Engagement, in this light, unfolds within a symbolic space of transition and negotiation—a *liminal condition*, in which individuals inhabit an existential threshold between dependence and autonomy, passivity and agency (Stenner and De Luca Picione, 2023; De Luca Picione, 2021). Appreciating this dialectic is essential for developing DHIs that are not only clinically effective but also psychologically resonant and responsive to the experience of living with a chronic illness.

This study aims to explore how individuals living with hypertension construct and attribute meaning to their engagement with digital self-monitoring technologies. Specifically, the research investigates the meanings that support the “retain” phase of McCarron et al.’s (2020) engagement model, asking: *Why do patients continue to be engaged?*

Adopting Braun and Clarke’s Reflexive Thematic Analysis (2006), this approach allows us to reflect on how digital tools contribute to the regulation of both self-care and engagement—particularly in how they mediate the individual’s relationship with the self, the illness, and healthcare providers. The present study adopts a clinical psychology perspective that interprets engagement as a meaning-making process that unfolds through narrative challenges (Lemmo et al., 2025b; Lemmo et al., 2025a), in which individuals redefine their relationship with self, their chronic condition, and the healthcare system—now mediated by digital monitoring technologies. These tools both organize behavioral patterns and modify personal narratives which affects how people understand their health status and develop self-control and interact with medical professionals. Through this lens, the study aims to offer clinically grounded insights into how digital technologies participate in sustaining engagement by shaping emotional regulation, therapeutic relationships, and the symbolic construction of self-care in everyday chronic illness management. Understanding these subjective mechanisms is essential not only for enhancing clinical outcomes but also for informing the design of more psychologically sustainable public health interventions in chronic care.

## 2 Materials and methods

The research followed COREQ (Consolidated Criteria for Reporting Qualitative Research) checklist to maintain transparency during all stages of design and data collection and analysis.

### 2.1 Study design

We employed a qualitative exploratory design using Reflexive Thematic Analysis (RTA; Braun and Clarke, 2006) to examine how individuals with chronic conditions experience and make sense of their engagement with digital health interventions (DHIs). The study was theoretically grounded in McCarron’s engagement framework and adopted a socio-constructivist epistemological stance, recognizing that meaning is co-constructed through language, context, and subjectivity (COREQ items 1–3).

### 2.2 Research team and reflexivity

The research team consisted of psychologists with expertise in clinical work and qualitative methodologies. The interviews were conducted by two researchers (Authors 1 and 2), who had no prior relationship with participants and introduced themselves as researchers investigating personal experiences of engagement with DHIs for self-care in hypertension (COREQ items 4–8). To enhance reflexivity and reduce interpretative bias, reflexive memos and peer debriefings were used throughout the research process.

### 2.3 Participant selection

This research was conducted as part of the HSMonitor project exploratory study aimed to evaluate the usability of novel digital solutions for tele-monitoring and disease self-monitoring in a cohort of patients from Hypertension center of Federico II University Hospital (F2UH; De Luca et al., 2021).

An innovative research-intervention model was implemented to identify the psychological and relational determinants of chronic illness adaptation across the lifespan.

The study participants consisted of adults diagnosed with hypertension who used advanced digital self-monitoring devices including a smart blood pressure watch and digital scale which their physicians prescribed for illness self-care engagement. The ICT-enabled solutions worked to enhance primary prevention alongside timely diagnosis and effective hypertension management. The smart blood pressure watch operated for on-demand or continuous blood pressure measurements while the smart scale automatically recorded weight data which it transmitted to the system. The mobile application received data from both devices through wireless connection to provide real-time feedback and track trends and share information with healthcare professionals.

The study employed purposive sampling to select participants who represented different sociodemographic groups and technology experience levels.

Participants were approached during routine clinical check-ups and were informed about the study’s aims, procedures, and voluntary nature. Of the 42 eligible patients contacted, 35 agreed to participate, while 7 declined due to time constraints or lack of interest.

The final sample consisted of adults diagnosed with hypertension who used digital self-monitoring devices. The gender distribution (30 men and 5 women) reflects the higher prevalence of hypertension among men in comparable clinical cohorts (World Health Organization, 2020), thus aligning with epidemiological trends in hypertension care.

Eligibility criteria included: (1) a diagnosis of hypertension for at least 1 year, (2) use of a digital self-monitoring device for at least 3 months, and (3) ability to participate in an interview in Italian. In total, 35 participants (30 men, 5 women; M age = 56.84, SD = 6.5; age range = 44–68) were recruited. Sampling continued until thematic saturation was reached (COREQ items 10–12, 22).

### 2.4 Setting and data collection

Participants were recruited through the same clinical setting described above. Semi-structured interviews were conducted either in person or via telephone, based on participant preference (COREQ items 14–15). The interview protocol included questions on engagement, emotional experiences, autonomy, and relational dynamics associated with DHI use (COREQ items 17–18). The interviews followed a narrative approach, moving from general to specific and aiming to elicit spontaneous, non-directive discourse. Their flexible structure was designed to preserve participants’ associative and discursive flow.

All interviews were conducted between March and July 2024, and held in an *ad hoc* room available for the interview. All interviews were conducted by the first and second authors, both clinical psychologists with experience in qualitative research and narrative methodologies. The shared gender and age range between interviewers and participants fostered trust and identification. A pilot interview round (*n* = 10) was conducted to refine the guide. Each interview lasted between 20 and 30 min, was audio-recorded, and transcribed verbatim. Field notes were taken to enrich contextual interpretation (COREQ items 19–20).

### 2.5 Ethical considerations

The study was approved by the Ethics Committee for Psychological Research of the Department of Humanities at the University of Naples Federico II (Approval number: 20/24). All participants gave written informed consent and were informed of their right to withdraw at any time. Personal data were anonymized in compliance with the EU GDPR 2016/679 and Italian Law D. L. 101/2018 (COREQ item 9).

### 2.6 Data analysis

The narrative material was analyzed using Reflexive Thematic Analysis (RTA) through the six-phase Braun and Clarke method (Braun and Clarke, 2021; Braun and Clarke, 2019; Braun and Clarke, 2006) to study subjective meaning-making processes situated within specific cultural contexts. The analytical process was guided by an inductive, bottom-up approach, meaning that themes were not pre-defined but emerged from the collected data (COREQ items 24–26). In line with this epistemological stance, the analysis proceeded through the following six phases: (1) familiarization with the transcripts, (2) generation of initial codes based on meaningful segments, (3) development of candidate themes, (4) review and refinement of themes, (5) naming and defining the final themes and subthemes, and (6) production of a final report that articulates the participants’ lived experiences and psychological dynamics. The two researchers D. L. and R. B. performed separate transcript reviews for multiple times to understand the data while creating initial manual codes without any predefined coding system. Throughout the analytic process, particular attention was paid to the emergence of a subtheme interpreted as a narrative reappraisal strategy of meaning-making, fundamental patterns within participants’ thoughts and emotional states and behavioral actions concerning digital health self-monitoring technology use. The team engaged in dialogical and reflexive discussions to develop shared meaning regarding the participants’ experiences while upholding their individual contextual backgrounds. All coding and thematic disagreements received resolution through team discussions and joint reflection sessions. The analytical process along with the thematic map received evaluation by F. M. who served as the third author to maintain methodological transparency and credibility. The thematic framework received iterative development to establish internal consistency and psychological depth and avoid redundant themes while maintaining relevant findings. The RTA approach did not require participant involvement for validating transcripts or findings because it focused on researcher involvement in knowledge co-construction rather than seeking intersubjective agreement (COREQ items 28–30). The use of RTA emphasizes the active, interpretative role of the researcher in the co-construction of knowledge (Braun and Clarke, 2019). Rather than aiming for inter-coder reliability through statistical measures, RTA acknowledges the value of reflexive engagement with the data and the analytic process. This methodological approach enabled us to examine the narrative challenges of the engagement process in Digital Health Interventions, focusing on the individual’s relationship with themselves, with the illness, and with the physician.

## 3 Results

The analysis produced three main themes which represent the meanings of the “retain” phase of engagement and the psychological and relational functions attributed to digital self-monitoring tools. Each theme responds to the core research question—why do individuals remain engaged— by highlighting a distinct dimension of meaning that sustains their continued use of these technologies in chronic illness management. The themes highlight how digital tools act as mediators between the individual and their self and illness and healthcare system. The Table 1 summarizes the overarching themes and their associated subthemes. Three Main themes of ‘Retain’ Phase Engagement in Digital Health Intervention for Self-care in Chronic Illness were identified.

**Table 1:** Main themes and subthemes of the “retain” phase of engagement in digital health interventions for self-care in hypertension.

Themes (*Why do I remain engaged?*)	Subthemes
1. Reassurance and sense of control over health status through digital tools	Making the invisible visible
	Encouraging active self-care management
	Providing a psychological anchor
2. Engaged but ambivalent and anxious: the emotional cost of monitoring	Compulsive monitoring vs. avoidance
	Desire to know vs. fear of knowing
	Emotional negotiation
3. Connected through data as numbers redefine the therapeutic dialog	Data as clinical dialog
	Feeling heard vs. feeling reduced to numbers
	Simulated presence

### 3.1 Reassurance and sense of control over health status through digital tools

This thematic category includes the meanings which describe the digital self-care monitoring device as a reliable companion in managing chronic illness—capable of offering immediate reassurance and fostering a greater sense of control. Through regular monitoring the device enables blood pressure detection which leads to immediate corrective actions that enhance the perception of active and competent self-management. This theme does not portray the device as a passive data collector, but rather as a promoter of self-awareness and health literacy, while becoming a fundamental element of daily routines. The monitoring process develops into a body-listening practice which helps people verify their condition’s stability through self-observation. The device transforms abstract risks into concrete measurable signals when managing invisible or asymptomatic conditions like hypertension by making the invisible visible.

*“Every morning I wake up, drink coffee, and measure my blood pressure. It's part of what makes me feel good.” (ID.4)*

*“Even if I feel good, I need to measure it to be sure. The number tells me if I'm really okay.” (ID.7)*

This sense of predictability and control allowed participants to anticipate problems, feel safer, and real-time decision-making abilities. Beyond its clinical utility, the device serves an emotional containment function, reducing anticipatory anxiety and strengthening a subjective sense of control over their condition.

*“When I see that the numbers are good, I can go out and feel more relaxed. If not, I take it easy.” (ID, 21)*

The theme contains three subthemes that explain the psychological and symbolic methods through which users develop their engagement.

- **Making the Invisible Visible**: The device creates visible measurements of an invisible health condition.
- **Encouraging active self-care management**: participants feel more capable of intervening preventatively or correctively.
- **Providing a psychological anchor**: the ritual of monitoring generates emotional containment and reassurance.

This theme demonstrates how Digital Health Interventions (DHI) deliver both improved clinical monitoring and mental clarity and reassurance which serve as active agents for subjective awareness while creating practical health management tools for daily life.

### 3.2 Engaged but ambivalent and anxious: the emotional cost of monitoring

This thematic category captures the ambivalent meanings individuals attribute to the act of digital self-monitoring. While the device offers comfort and predictability, it also introduces emotional tension, transforming engagement into a psychologically charged experience. The answer to why do I remain engaged? here lies in a paradox: monitoring offers a sense of safety, yet simultaneously generates anxiety, making engagement a fluctuating process of emotional negotiation rather than a stable behavior.

Participants described how the reassuring function of the device could shift into hypervigilance, dependence, or even emotional avoidance. Monitoring, rather than being empowering, sometimes became overwhelming—amplifying worries instead of containing them. For some, the urge to measure was compulsive; for others, the fear of results led to avoidance:

*“I used to check it five times a day. I couldn’t stop. Then I started to get more anxious than before because if I saw a bad number, I would panic. I knew it was not always reliable, but I still believed it completely.” (ID.13)*

*“There were days I didn’t want to see the number. I was afraid it would be high and ruin my day.” (ID.2)*

These narratives illustrate that engagement is not a neutral or purely rational process—it is deeply emotional, marked by an internal conflict between the need for control and the fear of loss of control. Some participants recognized that the act of measuring itself became a stressor:

*“Having to take the measurements made me anxious. At some point, I had to stop because I realized that even the value itself was being affected by my anxiety.” (id.28)*

This theme reveals that digital tools can expose emotional vulnerabilities, especially in individuals who feel unprepared to interpret or regulate the data autonomously. While the device makes the invisible visible, it may also amplify a sense of fragility—externalizing health into numbers that can overshadow the lived bodily experience. Engagement is therefore sustained not only by reassurance but also by emotional ambivalence: curiosity and control coexist with fear and insecurity. The theme unfolds through three subthemes that clarify how this emotional engagement is maintained:

- **Compulsive Monitoring vs Avoidance**: The device becomes either a source of compulsive checking or something to be avoided entirely.
- **Desire to Know vs Fear of Knowing**: Individuals feel torn between needing information and dreading the emotional impact of the results.
- **Emotional Negotiation**: Engagement reflects a continuous negotiation of emotional states, rather than a fixed or linear behavior.

This category demonstrates that sustained engagement is not always adaptive or linear—it is shaped by ambivalence, emotional coping, and the symbolic meanings individuals assign to their relationship with health and uncertainty.

### 3.3 Connected through data as numbers redefine the therapeutic dialog

This thematic category explores how digital self-monitoring reshapes the patient-provider relationship, transforming not only communication patterns but also the patient’s perceived role within the healthcare system. The device enabled patients to link the personal health observations with clinical appointments through its data collection and recall functions that provided both improved accuracy and patient autonomy:

*“Now I bring my numbers to the doctor. It helps me explain what happened.” (ID. 9)*

*“I feel like the doctor listens more when I show the measurements. Now the doctor looks at the numbers more than at me.” (id.17)*

The process of monitoring transformed patient symptoms from personal stories into quantifiable medical data which sometimes replaced subjective narratives altogether. The device enables patients to make their bodies quantifiable through numbers which creates a shared medical terminology for healthcare discussions. Patients who had hypertension benefited from the device since it gave them an established method to describe their body condition when symptoms presented themselves as unclear or hidden. The transition brought emotional side effects into play. The medical decision-making process became clearer to some participants, and they gained more involvement in healthcare choices because of this change.

*“They include me in their work like I’m one of them. I can follow the trend. I understand more now.” (ID. 22)*

Digital tools created a dual impact by providing patients with enhanced medical awareness yet establishing a feeling of distance in their relationship with healthcare providers. People could change their medication through phone calls to doctors when statistical abnormalities were detected.

The implementation of digital tools created a dual effect by allowing better medical information sharing and speedier interventions yet resulting in a sense of detachment between patients and healthcare providers. The quantifiable metrics transformed illness narratives which were originally expressed through words and sensations into numerical data.

The dual nature of this phenomenon appeared in how people understood the role of the device as either an expanded medical resource that mimicked provider contact between visits or as an electronic replacement for human connection. The device provided medical information while simultaneously functioning as a tool to preserve healthcare system connectivity through technology-based means. Patients sustained their engagement because they received assurance from monitoring and developed confidence from receiving information and felt a symbolic connection to their providers.

The category unfolds through three subthemes:

- **Data as Clinical Dialog**: The device facilitates communication with healthcare providers, translating subjective experience into a shared medical language.
- **Feeling Heard vs. Feeling Reduced to Numbers**: Engagement fluctuates based on whether the data enhances mutual understanding or replaces relational presence.
- **Simulated Presence**: Notifications, alerts, and data feedback are experienced as extensions of medical oversight, offering reassurance but also reconfiguring intimacy.

The medical oversight system delivers simulated presence through notification alerts and data feedback which provides reassurance but transforms intimacy in the process. Digital self-monitoring technologies transform both the emotional aspects and relational aspects of healthcare delivery which leads to new definitions of patient engagement through the combination of visibility with empowerment and human interaction.

## 4 Discussion

The findings of this qualitative study revealed three main thematic categories through which patients with hypertension interpret their engagement during the “maintenance” phase in the use of digital self-monitoring devices: (1) Reassurance and Sense of Control Over Health Status through Digital Tools (2) Engaged but Ambivalent and Anxious: The Emotional Cost of Monitoring (3) Connected Through Data as Numbers Redefine the Therapeutic Dialog. These themes clearly express the inner motivations—cognitive, emotional, and relational—that sustain long-term engagement.

The first theme shows that engagement with digital devices for hypertension is maintained through the device’s perceived ability to offer immediate reassurance, emotional stabilization, and a sense of control. These motivations respond directly to the core research question: people remain engaged because the device offers clarity amidst uncertainty, enabling symptom interpretation, anxiety reduction, and active health management.

The second theme indicates that engagement with DHIs can persist despite psychological discomfort, and that monitoring behaviors are not always indicative of well-being or empowerment. This theme shows that engagement can be emotionally mixed and sometimes causes anxiety. The process of digital monitoring leads people with hypertension to check their devices compulsively, creating emotional dependence and avoidance behaviors. The continuous presence of the device may even exacerbate health-related distress (Ojike et al., 2016), leading to hypervigilance and oscillation between the desire to know and the fear of knowing—exposing a fundamental conflict between safety and insecurity.

The first two thematic categories demonstrate how digital engagement in self-care management for hypertension patients create complex, non-linear emotional responses. These categories exist as opposite poles of a continuous engagement experience, not as mutually exclusive states. Engagement is therefore an emotional trajectory that fluctuates between reassurance and emotional exhaustion depending on specific circumstances.

These categories may also be shaped by emotional fluctuations, disease stages, prior experiences, and personal health meanings. This duality challenges the assumption that digital engagement is always positive or linear. On the contrary, it suggests that maintaining engagement requires a delicate psychological balance: the device offers structure and visibility, but can also externalize vulnerability and amplify distress—especially when patients feel unable to autonomously interpret the data.

This ambivalence echoes Schermer (2009) notion of the tension between autonomy and dependence in telemedicine. While digital devices provide structure and empowerment, they may also foster psychological dependence and distress. Similarly, Brimmel et al. (2023) emphasize that digital tools can temporarily regulate affect, but risk causing emotional overload or avoidance if not embedded within a broader emotional strategy. Recent models of digital affect regulation (Bettis et al., 2021) further emphasize how individuals use self-monitoring technologies to manage emotional arousal and uncertainty. However, such regulation can oscillate between adaptive emotional awareness and maladaptive vigilance, depending on how feedback and self-tracking are psychologically integrated.

The third theme, in response to the question “*Why do I remain engaged*?,” suggests that relational safety plays a central role. The device not only delivers health-related information but also becomes a medium for maintaining a—albeit mediated—connection to the healthcare system. Engagement is supported by the reassurance of being monitored, the feeling of being informed, and the symbolic sense of an ongoing clinical presence. This category, which focuses on how digital tools redefine the relationship with healthcare providers, highlights the symbolic and functional impact of data flows within the patient–clinician dynamic.

Participants developed a key understanding: real-time, personalized notifications represent a vital element in the interpretive process. Alerts are perceived as direct messages from the healthcare system, supporting self-monitoring while blending surveillance with autonomy. Notifications act as digital representations of clinical supervision, giving patients the feeling of being constantly cared for despite physical absence. Algorithmic signals expand the therapeutic bond into a data-based relationship that extends care from clinical appointments into everyday life. Many patients reported increased engagement and trust due to the continuous connection, while others expressed concerns around surveillance, emotional exposure, and responsibility. Healthcare providers use notifications as relational regulators to shape patient expectations and sustain behavioral and emotional patterns in chronic disease management. These digital outputs hold psychological meaning as they sit between feeling connected to others and feeling controlled by them—between reassurance and dependence.

These thematic categories reflect a spectrum of psychological experiences that both confirm and complicate existing literature. For instance, the idea that digital tools can enhance agency and safety in self-care aligns with findings by Morton et al. (2022), who emphasize how health technologies promote body awareness and self-regulation. However, our findings show that reassurance often coexists with anticipatory anxiety and hypervigilance, confirming Schermer (2009) concerns about the fragile balance between autonomy and dependence in tech-mediated care. The transformation of the patient–provider relationship also emerged clearly: while some participants appreciated the ability to share objective data, others reported a loss of emotional closeness—raising the risk that digitalization fragments clinical intimacy (Lupton, 2014; Coe et al., 2021).

Overall, these themes suggest that digital self-monitoring devices serve a regulatory function across three interconnected domains:

- Self-regulation: offering reassurance, structure, and bodily signal interpretation.
- Illness regulation: making chronic conditions more trackable, predictable, and manageable.
- Relational regulation: reshaping the experience of closeness and communication with care providers.

These patterns align with an iterative and situated model of engagement (Lemmo et al., 2025b; Lemmo et al., 2025a), where continuity is not only built through adherence, but through the symbolic integration of technology into one’s illness narrative.

**Figure 1:**
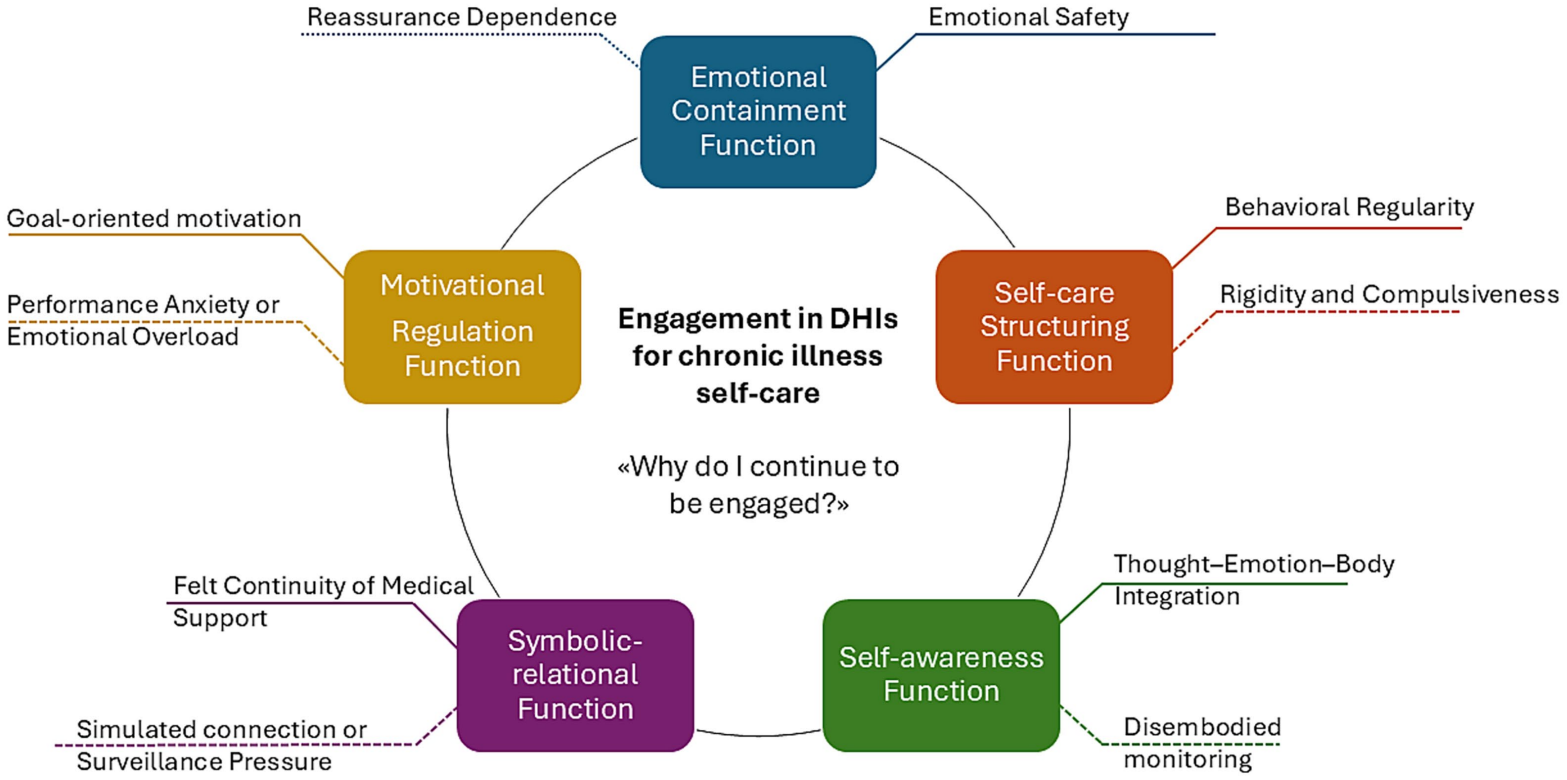
The Psychological functions and ambivalences of engagement in digital health intervention. This figure illustrates the main psychological functions that sustain engagement in Digital Health Interventions (DHIs) for self-care in chronic illness: Emotional Containment, Self-Care Structuring, Self-Awareness, Symbolic-relational, and Motivational Regulation. Each function is represented along a continuum, with a solid line indicating its functional expression (e.g., safety, regularity, integration, continuity, motivation) and a dashed line indicating its non-functional or ambivalent expression (e.g., dependence, compulsive behavior, disembodied monitoring, surveillance, and performance anxiety). Engagement emerges as a dynamic, iterative and situated process shaped by the tension between supportive and distressing experiences in the use of digital self-care monitoring devices.

Devices do not function as neutral data collectors—they emerge as psychologically mediating technologies that shape how people understand, manage, and emotionally respond to chronic illness in daily life. As previously highlighted by Lupton (2014) and Coe et al. (2021), digital tools support routines, symptom awareness, and uncertainty management. Our findings expand this view: digital devices are not only behavioral aids, but symbolic actors in patients’ engagement narratives. The research findings from this study expand existing knowledge about engagement models by two key aspects. The PHE Model (Graffigna and Barello, 2017) and the DIEGO Framework (O’Connor et al., 2016) both focus on patient traits and emotional elaboration and identity transformation in health engagement but our findings extend current theorization in two significant ways. The framework demonstrates how DHIs create emotional ambivalence through situated and fluctuating engagement experiences which depend on symbolic and emotional interpretations of the same technological interaction. The DIEGO Framework emphasizes contextual and motivational factors in digital health adoption but its main focus lies in recruitment and adherence mechanisms. The research extends this perspective by analyzing psychological factors which maintain long-term engagement (retain phase) and demonstrates how devices function as emotional regulators and symbolic mediators and relational anchors. The proposed framework develops a dynamic and processual and clinically-grounded understanding of digital engagement which serves both psychological theory development and person-centered DHI design. From a clinical psychology perspective, these devices may be conceptualized as technological co-regulators—tools that help contain and integrate illness-related anxiety while sustaining engagement over time. Many participants described real-time personalized notifications—such as alerts or reminders—as sources of emotional containment. These notifications not only inform patients, but also structure the self-care experience by offering immediate validation, uncertainty reduction, and perceived care continuity. These tools can also be understood as mediators between subjective bodily experience and external clinical frameworks. They help bridge the tension between autonomy and dependence, between experiential uncertainty and interpretive clarity. In this sense, the device is not simply a monitor, but a companion in the co-construction of meaning—actively involved in the everyday negotiation of illness, identity, and care. The findings suggest that people remain engaged with digital technologies not only for their clinical utility but because the engagement process in DHIs support core psychological functions (see Figure 1). The same functions that provide benefits can also produce psychological costs if not integrated into a broader emotional regulation strategy. For instance:

- ***Emotional containment function***

*General description.*

This function describes the role of engagement with self-monitoring devices as a form of emotional anchoring during periods of uncertainty or illness-related distress. Through data availability and structured interactions engagement helps users feel secure and emotionally calm thus enabling better health-related ambiguity navigation.

*Functional expression—emotional grounding.*

Through adaptive engagement individuals find emotional containment which creates an assurance framework while providing self-calming techniques and maintaining emotional equilibrium through regular monitoring practices. The experience creates feelings of security along with continuous medical care while providing individuals with control over their healthcare.

*Non-functional or ambivalent expression —reassurance dependence.*

Excessive engagement results in dysfunctional behavior which produces compulsive checking and hypervigilance as well as excessive reliance on digital reassurance. People who develop emotional dependency on technology become more susceptible to emotional distress when data shows fluctuations or difficulties in interpretation.

- ***Self-care structuring function***

*General description.*

This function describes the role of engagement with self-monitoring devices to support consistent behavioral patterns for managing chronic illness management. Digital routines that are structured help users maintain adherence to their care plan and build daily habits while creating predictable self-care practices.

*Functional expression— behavioral regularity.*

The adaptive use of engagement leads people to adopt consistent health-promoting practices. Through tracking activities individuals develop organized routines which enable them to manage diseases independently while feeling more autonomous.

*Non-functional or ambivalent expression— rigidity and compulsiveness.*

Maladaptive engagement produces rigid and inflexible scheduling patterns which trigger compulsive actions and distress when plans get interrupted. The rigid structure limits spontaneous actions while intensifying performance-related stress which weakens adaptive coping responses during unanticipated situations.

- ***Self-awareness function***

*General description.*

This function describes the role of engagement with self-monitoring devices to develop awareness about bodily sensations together with emotional states and cognitive patterns and behavioral responses. This function allows people to develop reflective understanding that helps them make informed choices about self-care through the connection between inner and outer signals.

*Functional expression—thought–emotion–body integration.*

People who use engagement effectively develop emotional-cognitive awareness because they can better observe the connections between their thoughts and feelings and physical symptoms and behaviors which leads to better self-regulation and understanding.

*Non-functional or ambivalent expression—disembodied monitoring.*

The dysfunctional manifestation of engagement produces fragmented and hyper-analytical interpretations of bodily signals that lack emotional connection. The separation between body and mind through disembodiment creates higher cognitive stress while increasing anxiety and making people feel detached from their physical self.

- ***Symbolic-relational function***

*General description.*

This function describes the role of engagement with self-monitoring devices to establish a felt relationship with healthcare providers or develop feelings of receiving proper care. Digital health tools enable patients to maintain symbolic relationships with the care system through notification systems and data sharing capabilities.

*Functional expression— felt continuity of medical support.*

Positive engagement creates relational continuity by making patients feel accompanied while increasing their sense of security and strengthening their connection to healthcare providers beyond scheduled appointments. The relationship between patients and healthcare providers strengthens through this approach which also motivates patients to follow treatment recommendations.

*Non-functional or ambivalent expression—simulated connection or surveillance pressure.*

The dysfunctional engagement substitutes actual human interaction with artificial presence which produces emotional isolation or performance-based expectations for the device. Users experience feelings of being under constant observation along with feelings of judgment and social detachment.

- ***Motivational regulation function***

*General description.*

This function refers to how engagement with digital self-monitoring devices sustains the motivation for health behavior change. The combination of immediate feedback with reminder alerts and visual progress tracking creates sustained commitment toward goal-oriented actions.

*Functional expression – goal-oriented motivation.*

Effective engagement provides motivational support which helps people achieve their goals while delivering positive reinforcement. The combination of focus and empowerment and health change initiation capabilities leads individuals to maintain health-related modifications successfully.

*Non-functional or ambivalent expression—performance anxiety or emotional overload.*

The excessive use of engagement transforms into performance pressure which causes people to feel anxious about their results while facing discouragement from unmet objectives. Excessive emotional weight on users diminishes their natural drive to participate and elevates their probability of abandoning the activity.

The psychological functions described in this study—such as emotional containment, self-care structuring, symbolic-relational, etc.—should not be interpreted as inherent characteristics of the digital interventions or of the devices themselves. These functions develop through the actual experience of engagement using digital tools which people shape through their personal interpretations, emotional responses and environmental elements. From this viewpoint engagement represents a complex psychological process which users experience during their device interactions while assigning personal value to their interactions. The same digital tool can thus fulfill different functions depending on the user’s psychological needs, illness trajectory, and relational context.

From this perspective, digital health tools operate as semiotic regulators—technologies that not only measure, but shape the meaning of care, illness, and selfhood. The device becomes a “third presence,” mediating the space between patient and clinician, between self and illness. It can contain anxiety and support meaning-making, but may also embody internalized pressures of performance and vigilance. Distinguishing between adaptive and maladaptive engagement becomes crucial: while digital tools can enhance agency, structure, and emotional containment, excessive reliance may produce digital distress—manifested as anxiety, guilt, or self-surveillance. Clarifying this boundary helps clinicians identify when engagement shifts from supportive to dysregulating. However, this very structure may carry a psychological cost. A crucial clinical question arises: when does containment become dependence? When does monitoring become hypervigilance? The same notifications that provide stability can trigger dependence and stress, particularly when patients feel compelled to remain constantly “connected” to maintain control over their condition.

The emotional burden of self-care may manifest as motivational ambivalence or low-grade chronic distress, ultimately threatening the sustainability of engagement. From this perspective, engagement should not be viewed as neutral or automatic behavior, but as a psychologically regulated process—marked by effort, emotional conflict, and continuous negotiation.

Clinicians should avoid interpreting usage frequency as a straightforward indicator of engagement. Instead, they should explore the emotional dynamics underlying self-monitoring and support patients in creating sustainable behavioral and emotional routines. Engagement should be approached as a process of care, not a performance or a form of surveillance.

Clinical engagement requires a new approach that recognizes its threefold nature: emotional, relational, and symbolic. Clinical psychologists must ask not only whether engagement is sustained, but at what psychological cost and through which internal dynamics.

Self-care in chronic illness is not merely adherence. It demands the capacity to reshape identity, master autonomy, and regulate emotions. Digital devices do not replace the therapeutic relationship—they now actively participate in it.

This research shows that effective engagement with digital technologies represents a psychological achievement, not an automatic process. While monitoring functions help patients manage their care journey, the true power of these tools lies in their ability to help construct meaning, emotional connection, and therapeutic continuity. Maintaining digital engagement requires emotional integration as much as it does technical connection.

Our findings align with and extend current literature on digital health experience in chronic illness.

Recent reviews have emphasized that experiences of digital self-monitoring are often ambivalent, combining reassurance and emotional burden (Taylor, 2022). This duality echoes the emotional fluctuations observed in our participants, who reported both empowerment and anxiety linked to continuous data visibility. As Johnsson et al. (2023) note, self-care monitoring represents more than behavioural tracking—it is an interpretive process through which patients negotiate bodily sensations, emotions, and meanings of illness in everyday life.

Our study advances this view by conceptualizing engagement as a psychological trajectory rather than a static behavioral state, characterized by emotional regulation, symbolic mediation, and relational negotiation.

Furthermore, evidence from Lu et al. (2025) shows that sustained engagement in digital self-care depends on motivational and emotional design features, such as personalized feedback and perceived relational support. These findings converge with our interpretation of digital devices as semiotic and affective regulators—technologies that help patients maintain psychological continuity in self-care while also risking dependence and digital distress.

In this light, digital monitoring should not be interpreted solely as a behavioral outcome but as a process of meaning-making that connects emotional containment, relational anchoring, and identity regulation throughout chronic illness trajectories.

### 4.1 Clinical and theoretical implications

This study offers several important implications for clinical practice, psychological theory, and the design of Digital Health Interventions (DHIs).

From a clinical psychology perspective, the findings suggest that digital engagement should be assessed not only in terms of adherence but also through its emotional and relational dimensions. Health professionals should recognize that usage frequency or compliance with protocols are not sufficient indicators of healthy engagement, as patients may appear highly active while simultaneously experiencing anxiety, dependence, or digital fatigue.

Regarding the ambivalent emotional response to continuous monitoring, health professionals and psychologists should introduce reflective moments in clinical conversations focused on emotions related to data—such as reactions to alerts or fluctuations—to help patients transform anxious control into reflective self-regulation and prevent the shift from reassurance to hypervigilance.

Clinicians can act as co-regulators of meaning, using digital data to promote dialog, emotional containment, and shared understanding. This perspective positions digital devices within, rather than outside, the therapeutic alliance.

DHIs should be evaluated not only for their effectiveness and clinical outcomes but also for their emotional and relational effects. Digital devices help patients contain illness-related anxiety and integrate data into the narrative construction of self and care. However, these tools can also amplify dependence and control, especially among more vulnerable individuals.

Digital notifications function as extended symbols of caregiving: they provide comfort and a sense of clinical presence but can also trigger unconscious emotions of surveillance or judgment. Understanding these dualities enables clinicians to better manage motivational ambivalence, emotional distress, and resistance to self-care.

Findings on emotionally intelligent technologies open new perspectives for the future design of such systems. Developers and designers of DHIs should consider the emotional and symbolic effects that their tools produce.

Two critical areas deserve priority in design:

- Regulation of hypervigilance: the risk arises when excessive monitoring combines with poorly calibrated feedback loops, generating anxiety, compulsive checking, and a sense of loss of control. Systems should be able to monitor users’ emotional responses and provide support without overwhelming their experience.
- Emotional safety in personalized notifications: messages should not only provide clinical information but also convey a sense of human connection. The use of empathic language, visual metaphors of stability, and self-regulation options can strengthen the sense of care and reduce distress.

This research broadens theoretical perspectives on engagement by showing that it develops through psychological processes emerging within technological interactions and linked to patients’ illness narratives.

Engagement should therefore be understood as a symbolic practice of care that connects self-regulation, affective containment, and relational continuity, rather than as a simple indicator of usage or adherence.

A conceptual shift is needed in how researchers and clinicians define and measure engagement, recognizing it as a relational and affective process.

By integrating emotional awareness into both clinical practice and technological design, digital health systems can move beyond mere behavior modification to support identity reconstruction, emotional resilience, and truly sustainable engagement.

Finally, this study contributes to the growing literature on the concept of technological alliance (Riva et al., 2019; Wiederhold, 2020), suggesting that digital devices can act as relational mediators within the therapeutic process. Incorporating these insights into clinical training may help psychologists and healthcare providers use DHIs not merely as monitoring tools but as extensions of the therapeutic relationship—fostering empathy, continuity, and emotional safety in digitally mediated care.

In sum, digital engagement is not a purely technological phenomenon but a relational and affective process. Recognizing its emotional costs and regulatory potential enables professionals to transform digital monitoring into a psychologically integrated practice of care.

### 4.2 Limitations

This study presents several limitations that should be acknowledged. First, the sample was predominantly male (30 men, 5 women), which may limit the transferability of the findings to a more gender-balanced population. Additional studies should investigate possible differences between genders when it comes to emotional control and Digital Health Intervention use.

The study participants came from a single outpatient clinic in Southern Italy thus their experiences could be influenced by both cultural elements and healthcare system characteristics. Additional research needs to be conducted across different cultural backgrounds to validate these findings for various demographic groups.

This research maintained reflexivity by using memos and peer debriefing, yet excluded participant involvement in validating the results through member-checking which might have reduced meaning co-construction. Future qualitative designs should include participant involvement during the interpretation phase to gain richer findings.

The research examined engagement specifically during the “retain” phase only. Research on the “recruit” and “sustain” phases would provide a better understanding of how engagement changes through time by connecting psychological states with technological interactions.

### 4.3 Conclusion

This study explored how individuals with chronic illness, specifically hypertension, experience and make sense of their engagement with digital self-monitoring tools.

Through reflexive thematic analysis we discovered three main themes which demonstrate how these devices operate both clinically and psychologically to manage emotions while establishing routines and transforming treatment relationships.

The study results contradict basic engagement models that rely solely on user’s usage metrics, highlighting instead the need to understand engagement as a social achievement which depends on emotional and relational factors. Digital tools function as “technological others” because they provide both supportive containment and tension and reassuring comfort and dependence along with presence and control functions. Digital tools assist daily self-care by reshaping how people interact with their medical conditions and their healthcare team and their personal power.

The study provides innovative paths for both clinical psychology and health technology design through its definition of engagement as a meaningful psychological process. Digital engagement requires clinicians to study emotional responses from patients while designers need to develop tools that fulfill both behavioral and emotional and symbolic requirements of users.

Sustaining digital health engagement exceeds basic user retention metrics because it requires more than continued use. The process includes creating emotional integration while developing narrative coherence and establishing a sense of connectedness. The complete realization of DHIs as person-centered sustainable care tools requires attention to the psychological elements of digital engagement.

## Data Availability

The original contributions presented in the study are included in the article/supplementary material, further inquiries can be directed to the corresponding author.
